# Synthesis and Characterization of Hybrid Molecularly Imprinted Polymer (MIP) Membranes for Removal of Methylene Blue (MB)

**DOI:** 10.3390/molecules17021916

**Published:** 2012-02-15

**Authors:** Saliza Asman, Nor Azah Yusof, Abdul Halim Abdullah, Md Jelas Haron

**Affiliations:** 1 Department of Chemistry, Faculty of Science, University of Malaya, Kuala Lumpur 50603, Malaysia; Email: saliza_asman@yahoo.com; 2 Department of Science and Mathematics, Faculty of Science, Technology and Human Development, Universiti Tun Hussein Onn Malaysia, Parit Raja 86400, Johor, Malaysia; 3 Department of Chemistry, Faculty of Science, Universiti of Putra Malaysia, Serdang 43400, Selangor, Malaysia; Email: halim@science.upm.edu.my (A.H.A.); mdjelas@science.upm.edu.my (M.J.H.); 4 Institute of Advanced Technology (ITMA), Universiti of Putra Malaysia, Serdang 43400, Selangor, Malaysia

**Keywords:** molecularly imprinted polymer (MIP), MB-MIP membrane, methylene blue (MB), cellulose acetate (CA), polysulfone (PSf)

## Abstract

This work reports the synthesis and characterization of a hybrid molecularly imprinted polymer (MIP) membrane for removal of methylene blue (MB) in an aqueous environment. MB-MIP powders were hybridized into a polymer membrane (cellulose acetate (CA) and polysulfone (PSf)) after it was ground and sieved (using 90 µm sieve). MB-MIP membranes were prepared using a phase inversion process. The MB-MIP membranes were characterized using Fourier Transform Infrared Spectroscopy (FTIR) and Scanning Electron Microscope (SEM). Parameters investigated for the removal of MB by using membrane MB-MIP include pH, effect of time, concentration of MB, and selectivity studies. Maximum sorption of MB by PSf-MB-MIP membranes and CA-MB-MIP membranes occurred at pH 10 and pH 12, respectively. The kinetic study showed that the sorption of MB by MB-MIP membranes (PSf-MB-MIP and CA-MB-MIP) followed a pseudo-second-order-model and the MB sorption isotherm can be described by a Freundlich isotherm model.

## 1. Introduction

Molecular imprinting is a convenient and powerful technique for preparing polymeric materials with artificial receptor-like binding sites for various substances [1]. The selectivity of the polymer depends on various factors such as the size and shape of the cavity and rebinding interactions [2]. Recently, the imprinting technique has been extended to polymeric membranes [3,4,5,6], which can separate a target molecule from a mixture solution by permeating through the thin membrane. The imprinted membranes have potential applications in several fields. This is due to the high possibility of such membranes, which have perm-selective binding to target molecules, being used as separation tools [7,8,9,10]. Separations with membranes do not require additives, and they can be performed isothermally at low temperatures compared to other thermal separation processes. Also, upscaling and downscaling of membrane processes as well as their integration into other separation or reaction processes is easy [11]. 

MIP membranes have been used in many applications, especially in optical sensors; detection of digoxin in serum samples [12], biomimetic sensors; detection of phenols [13], screening of complex mixtures of haloacetic acids in drinking water [14], chloramphenicol succinate in milk [15] and salbutamol in pig urine [16]. The polymeric membranes could separate the target molecule(s) from the solution mixtures by selective permeation through the thin membrane [17]. Membrane technology has been already applied in many industrial fields (e.g., food [18,19], energy [20], environment [21], artificial organs) but, the possibility of introducing specific recognition sites in a synthetic membrane plays an important role for the transport of specific substances. An imprinted membrane is able to discriminate between target molecules and others, thus improving the separation process. 

According to previous reports, a phase inversion process has been used as a fabrication method for MIP membranes [17,22,23,24,25,26,27,28]. The MIP membrane made by using phase inversion processes has shown recognition and binding of the target molecule in continuous permeation operations in treating large amounts of solute solution. It is believed that this kind of membrane offers a promising technology for the selective binding to environmental disruptor targets [23]. 

In this work, cellulose acetate (CA) polymer and polysulfone (PSf) polymer were selected as support membranes in the preparation of MB-MIP membranes. CA polymer is a chemically modified form of cellulose which can be obtained in a variety of forms such as fibers, flakes and films. The application of CA in many fields is probably due to the significant number of unacetylated hydroxyl groups which permits higher water content at saturation with stronger hydrogen bonding, which is suitable for aqueous and high polar solvents and it also easily bonds with plasticizers. PSf polymer is a thermoplastic polymer that contains aryl-SO_2_-aryl sub-units which define the sulfone group feature. PSf is stable towards high temperatures, has high physical strength and a transparent surface. It is also stable in acids, bases and non-polar solvents. 

Dyes are constituents that are widely used in the textile, paper, plastic, food and cosmetic industries [29]. Textile printing is an important method for decorating textiles. Cellulosic fibers are the most commonly printed substrate and reactive dyes are, by far, the most commonly used colorants in textile printing. There are more than 100,000 commercially available dyes with over 7 × 10^5^ tonnes of dyes being produced annually. About 1,000 L of water is used for every 1,000 kg clothes processed in the dyeing section of a textile industry [30].

Many types of adsorbents are available for removal of dyes such as activated carbon and silica [31], however, their operating costs are high [32]. Catalysts like TiO_2_ have great adsorption capacity for organic compounds, but they have many limitations such as the recollection and reuse [33]. Adsorption by agricultural waste and by-products are the most widely used for removal of dyes because of its low initial cost, simplicity of design, ease of operation, and insensitivity to toxic substances, but the qualifications of the selectivity and specificity properties are very limited. The reusability of the products had not been reported. 

**Figure 1:**
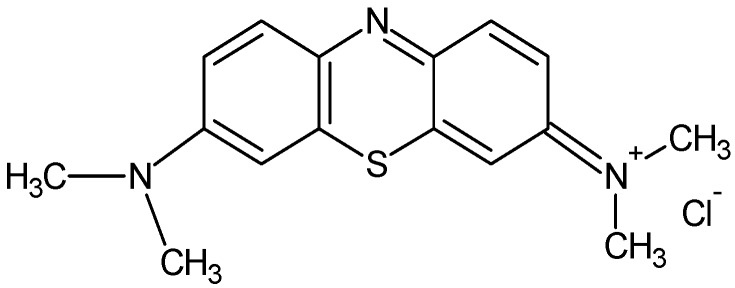
Chemical structure of MB.

In the present study, MB-MIP membrane was used as sorbent for the removal of cationic dye from aqueous solution. MB (Figure 1) was selected as a model compound in order to evaluate the capacity of MIP membrane for the removal of dyes from aqueous solutions. The aim of this study was to investigate the adsorption of MB into MIP membrane, which is a specific and selective sorbent for the removal of dyes and inexpensive for operating costs.

## 2. Results and Discussion

### 2.1. Characterization of MB-MIP Membranes Using FTIR

**Figure 2:**
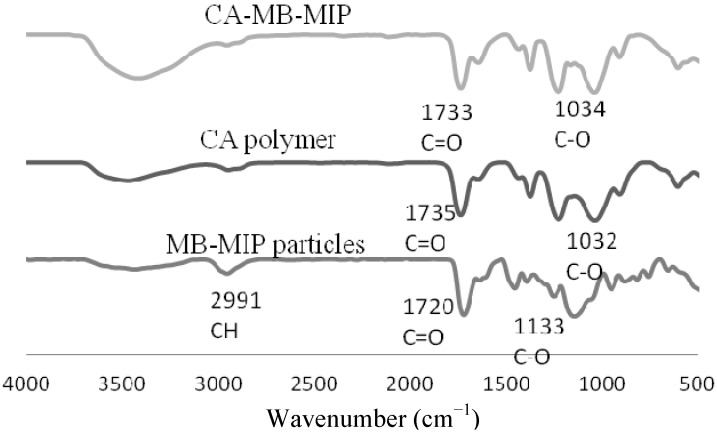
IR spectra for CA-MB-MIP membranes, CA polymer and MB-MIP particles.

The IR spectra of MB-MIP powders, CA polymer and CA-MB-MIP membrane are shown in Figure 2. The IR spectrum of CA polymer indicates that the stretching vibrations at 1,735 cm^−1^ and 1,032 cm^−1^ which correspond to C=O and C–O stretching vibrations, respectively, shift a little to 1,733 cm^−1^ and 1,034 cm^−1^ in the CA-MB-MIP membrane IR spectrum. These results confirm the hybridization process between MB-MIP with CA polymer. 

**Figure 3:**
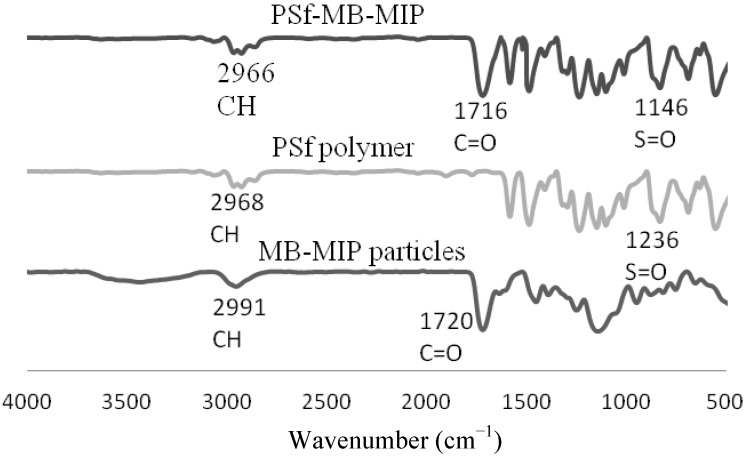
IR spectra for PSf-MB-MIP membranes, PSf polymer and MB-MIP particles.

Figure 3 shows the IR spectra of PSf-MB-MIP membranes, PSf polymer and MB-MIP particles. The absorption bands at 2,966 cm^−1^, 1,716 cm^−1^ and 1,146 cm^−1^ peaks are assigned to the C–H, C=O group and S(=O)_2_ groups, respectively. There is a slight shift for the sulfonyl group S(=O)_2_ peak in the PSf IR spectrum. The shift is probably due to hybridization of MB-MIP into the PSf membrane. The same observation has been made by Takeda *et al*. when using a hybrid membrane for indole derivatives and bisphenol derivatives [23].

### 2.2. Characterization of MB-MIP Membranes Using SEM

**Figure 4:**
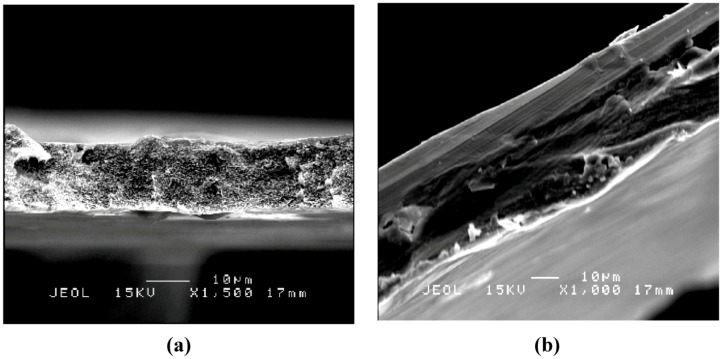
Cross section of (**a**) CA-MB-MIP membrane and (**b**) CA membrane.

**Figure 5:**
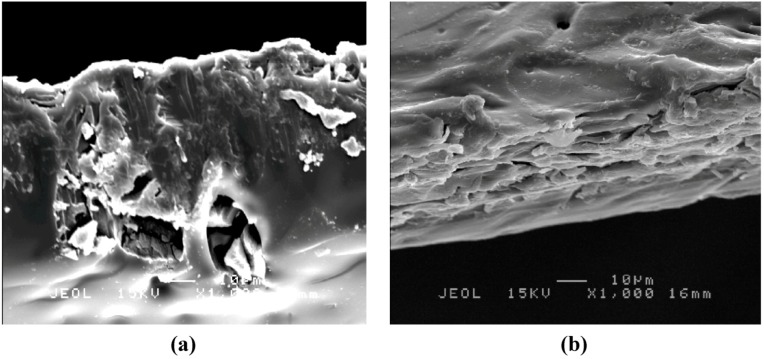
Cross section of (**a**) PSf-MB-MIP membrane and (**b**) PSf membrane.

Figure 4 and Figure 5 show SEM images of a cross-section of the CA-MB-MIP membrane (Figure 4a) and PSf-MB-MIP membrane (Figure 5a) and the corresponding non-imprinted membranes (Figure 4b and Figure 5b), respectively. From the SEM images, we note that the MB-MIP powders were successfully hybridized inside the polymer membranes, whereas the non-imprinted membranes show a smoother cross-section surface area. The morphological analysis also indicated that the CA-MB-MIP membrane has a more porous structure compared to the PSf-MB-MIP membrane. 

### 2.3. Effect of pH for Sorption of MB Using MB-MIP Membranes

**Figure 6:**
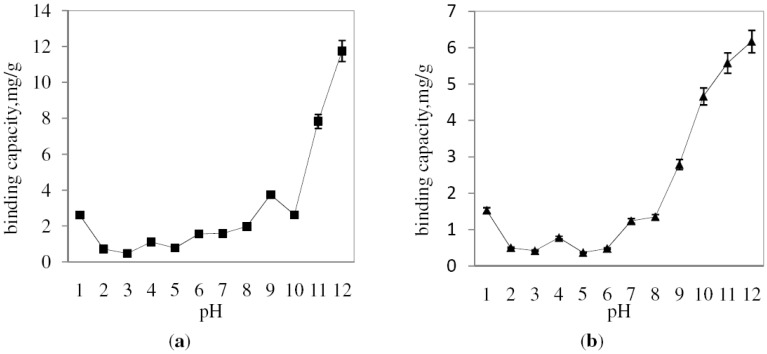
pH study for sorption of MB using CA-MB-MIP (**a**) PSf-MB-MIP (**b**) membrane.

Figure 6a and 6b showed the binding capacity of MB on CA-MB-MIP membrane and PSf-MB-MIP membrane at various pH values, respectively. The sorption of MB into the CA-MB-MIP membrane is low at acidic pH and started to increase at pH 7. A similar trend is observed for sorption of MB on PSf-MB-MIP membrane. It is suggested that pH affects the surface charge of the adsorbents, as well as the degree of ionization of the dye. As the pH increases, it is expected that the dye sorption will increase due to increasing negative surface charge of the adsorbents. 

### 2.4. Kinetic Study for Sorption of MB using MB-MIP Membranes

**Figure 7:**
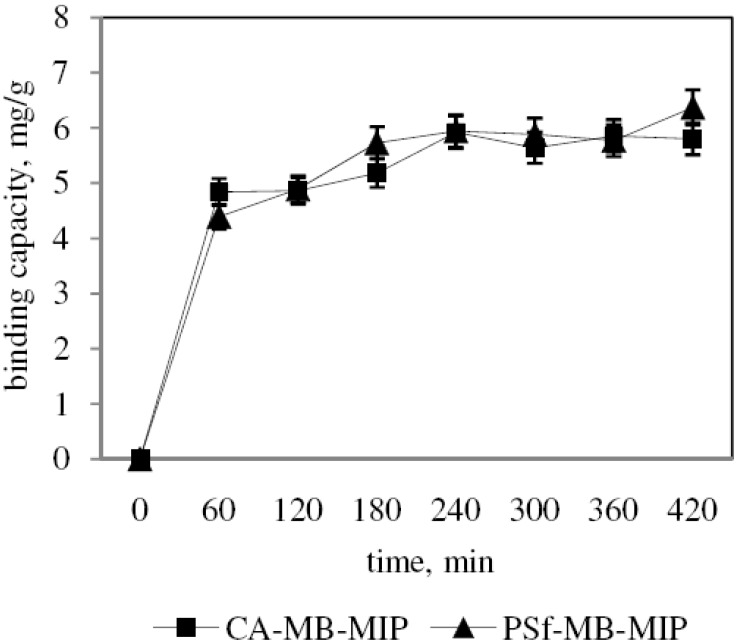
Kinetic study for sorption of MB on CA-MB-MIP membrane and PSf-MB-MIP membrane.

The experimental results of our kinetic study on sorption of MB are shown in Figure 7. The experiments were carried out at room temperature (25 °C) with optimized pH. The results indicate that sorption of MB using CA-MB-MIP membrane increased rapidly during the first 60 min and equilibrium was achieved at 180 min. A similar model is also was observed for sorption of MB using PSf-MB-MIP membrane. Ong *et al.* [29] explained that the dynamic adsorptive separation of solute from solution onto adsorbents depends on a good description of the equilibrium separation between the two phases. It is based on the assumptions that the maximum sorption correspond to saturated monolayer of sorbents on the adsorbent surface until the energy of sorption is constant and no migration in the plane of the surface occurs.

Rate constants for each membrane are summarized in Table 1. By following the pseudo first order model, the k values and correlation coefficient, R^2^ values based on CA-MB-MIP membrane and PSF-MB-MIP membrane are calculated to be 0.0064 min^−1^ (0.7280) and 0.0018 min^−1^ (0.7535), respectively, whereas with a pseudo second order model, the R^2^ values obtained are 0.9950 and 0.9906 for CA-MB-MIP membrane and PSf-MB-MIP membrane, respectively. The pseudo second order model gives q_e_ for sorption of MB on CA-MB-MIP membrane of 5.9737 mg/g and PSf-MB-MIP membrane of 6.3492 mg/g, which are similar to the experimental values of 5.9200 mg/g and 6.3700 mg/g respectively. It is suggested thus that the kinetic studies for MB-MIP membranes agreed well with a pseudo second order model. The pseudo second order can be applied for the entire sorption process and described the chemosorption of MB on the MB-MIP membranes. The rate limiting step may be chemical sorption involving valency forces through sharing or exchange of electron between sorbent and sorbate [34].

**Table 1:** The rate constant and value of binding capacity, q_e_ for the sorption of MB dye solution.

**Membranes**		**Pseudo first order kinetic model**			**Pseudo second order kinetic model**		
	q_e_ exp,	R^2^	q_e_,	k	R^2^	q_e_,	K
	(mg/g)		(mg/g)	(mg/g·min^−1^)		(mg/g)	(mg/g·min^−1^)
CA-MB-MIP	5.9200	0.7280	1.7640	0.0064	0.9950	5.9737	0.0123
PSf-MB-MIP	6.3700	0.7535	1.2503	0.0018	0.9906	6.3492	0.0080

### 2.5. Isotherm Study for Sorption of MB Using MB-MIP Membranes

**Figure 8:**
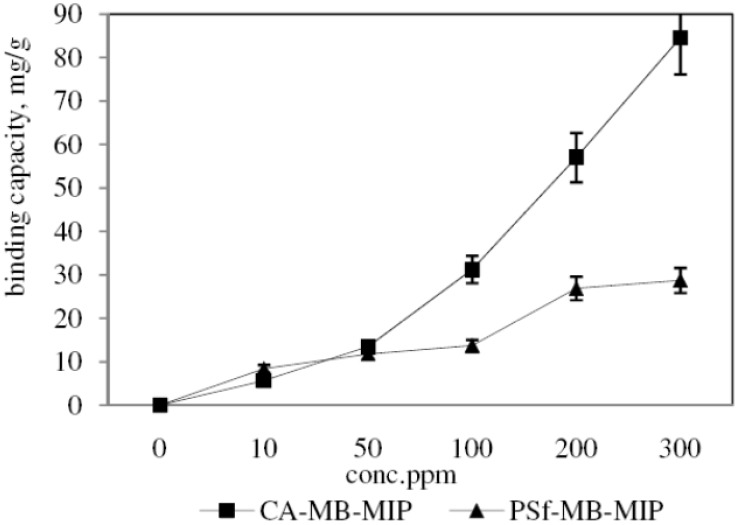
Sorption isotherms for the sorption of MB on CA-MB-MIP membrane and PSf-MB-MIP membrane.

Figure 8 shows the dependence of the equilibrium concentration of MB on the sorption rate of both membranes, CA-MB-MIP and PSf-MB-MIP membrane. The sorption value increases with increasing concentration of MB solution and the maximum sorptions for the CA-MB-MIP and PSf-MB-MIP membrane are 84.56 mg/g and 28.72 mg/g respectively. It can be concluded that CA-MB-MIP membrane adsorbed MB better than PSf-MB-MIP membrane. 

The Langmuir and Freundlich isotherm models are employed to describe the MB sorption process. The calculated values for the correlation coefficient, R^2^ and sorption constant values of these two models are shown in Table 2. R^2^ values for the Langmuir isotherm model for the sorption of MB on CA-MB-MIP and PSf-MB-MIP membrane are 0.4357 and 0.6779, respectively, whereas the R^2^ values for the Freundlich isotherm model for the sorption of MB on CA-MB-MIP and PSf-MB-MIP membrane are 0.9025 and 0.9820, respectively. The isotherm sorption process is likely to be modeled as a Freundlich isotherm based on the R^2^ values. The Freundlich isotherm model gives the relationship between equilibrium liquid and solid phase capacity based on multilayer adsorption [35]. 

**Table 2:** Langmuir and Freundlich constants for the sorption of MB dye solution.

Membranes	Langmuir isotherm model			Freundlich isotherm model		
	R^2^	q_e_, (mg/g)	b (L/mg)	R^2^	n	K_f_
CA-MB-MIP	0.4357	119.0476	0.0062	0.9025	1.5251	1.8463
PSf-MB-MIP	0.6779	43.1034	0.0080	0.9820	1.1258	0.9940

### 2.6. Selectivity Study of the MB-MIP Membranes

**Figure 9:**
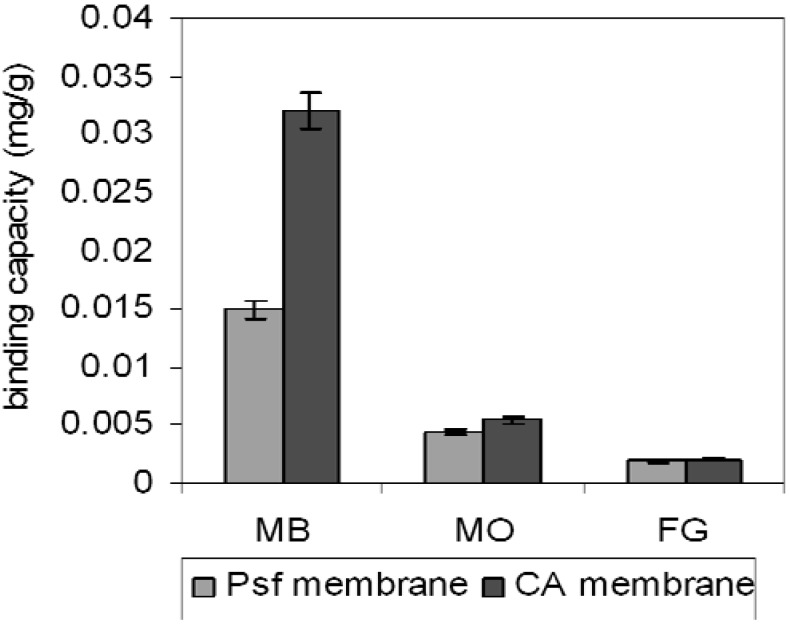
Selectivity studies of CA-MB-MIP membrane and PSf-MB-MIP membrane.

In order to study the selectivity properties of MB using MB-MIP membranes, the binding capacities of both membranes towards corresponding dye solutions including methylene blue (MB), methylene orange (MO) and fast green (FG) are shown in Figure 9. 

**Figure 10:**
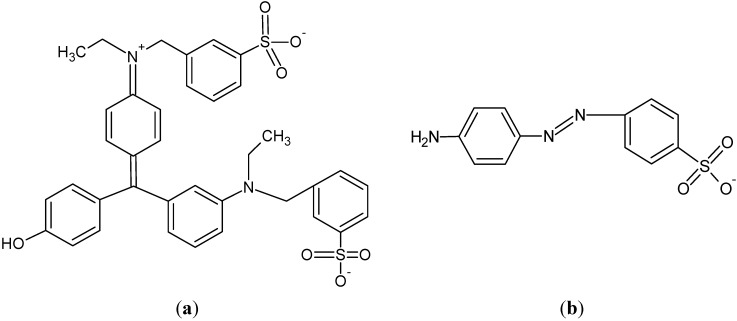
(**a**) Methylene orange and (**b**) Fast green chemical structures.

The binding capacities of MB, MO, and FG using the CA-MB-MIP membrane are 0.032 mg/g, 5.43 × 10^−3^ mg/g and 2.08 × 10^−3^ mg/g, respectively, whereas the binding capacities for MB, MO and FG using PSf-MB-MIP are 0.015 mg/g, 4.39 × 10^−3^ mg/g and 1.88 × 10^−3^ mg/g, respectively. These results suggest that both MIP membranes have higher sorption binding capacities for MB than other dyes. The recognition site of MB-MIP probably attracts the MO (Figure 10a) by electrostatic interaction caused by the amine group in its structure similar to MB. However, for FG (Figure 10b) the sorption is very low, due to non-similar structure compared to MB. It can be concluded that, the prepared MB-MIP membranes are selective towards MB. We have also compared the selectivity of the synthesized MIP and non-imprinted polymer (NIP) (reported elsewhere [36]), where the synthesized MIP has shown greater binding capacity towards methylene blue compared to NIP.

## 3. Experimental

### 3.1. Materials

Methylene blue (MB), methacrylic acid (MAA), benzyol peroxide (BPO) and tetahydrofuran (THF) were supplied by R&M Chemical (Selangor, Malaysia). Ethylene glycol dimethacrylate (EGDMA) was supplied by Sigma-Aldrich Chemical (Steinhem, Germany). Polysulfone (PSf) was supplied by Acros Organics (Geel, Belgium) and Cellulose Acetate (CA) was supplied by BDH Chemicals (Selangor, Malaysia) All other materials were of reagent grade and purchased from Merck (Steinhem, Germany). Distilled water was used throughout the experiment. 

### 3.2. Instruments

The sorption experiments were studied using Varian-Cary Win UV 100 spectrophotometer (Shimadzu, Kyoto, Japan). The MIP membranes were characterized by Fourier Transform Infrared (FTIR) spectroscopy using a Perkin-Elmer 1600 Spectrophotometer (New York, NY, USA) and a Scanning Electron Microscopy (SEM) JEOL JSM 6400 (Tokyo, Japan).

### 3.3. Experimental

#### 3.3.1. Preparation of MB-MIP Powders

MB dye (0.12 mmol) was dissolved in MAA (0.93 mmol) and EGDMA (4.65 mmol) solution. MB-MIP powders were obtained using a molar ratio of 5:1 (crosslinker/monomer). The solution was then transferred to a mixture containing BPO (10.0 mg) and THF (30.0 mL). Immediately, the mixture was purged with nitrogen gas for 10 min to remove the oxygen. The solution was sealed and placed in a water bath for overnight. The resulting MIP polymer powders were ground and sieved to 90 µm before extraction, then the template was then removed from the polymer by washing with methanol/acetic acid/water mixture (8:1:1 ratio) until the template was removed completely.

#### 3.3.2. Preparation of the MB-MIP Membranes

MB-MIP membrane was prepared using cellulose acetate and polysulfone. MB-MIP (0.05 g) was mixed with each polymer solution (1.00 g) and the mixture was stirred at 50 °C until a homogeneous solution was obtained. Herein, acetone (30.00 mL) and THF (30.00 mL were used as solvents for the CA polymer and PSf polymer, respectively. The resultant mixture was spread on a glass plate with 100 µm thick spacer at 50 °C, and dried at room temperature. Non-imprinted hybrid membranes were prepared without using MB-MIP in PSf and CA. The MB-MIP membranes were characterized using FTIR and SEM analysis.

#### 3.3.3. Effect of pH for Sorption of MB using MB-MIP Membranes

The pH of MB solution (10 ppm) was adjusted by adding diluted HCl or NaOH. Subsequently, the prepared solution (20.00 mL) was shaken with a piece of MB-MIP membrane weighing 0.012 g. The membrane was incubated in MB solution at room temperature for 1 h. The filtrate of the MB solution was analyzed and the optimum pH for maximum sorption was determined.

#### 3.3.4. Kinetic Study for Sorption of MB using MB-MIP Membranes

A piece of MB-MIP membrane (0.012 g) was added to 10 ppm MB solution (20.00 mL) and stirred for different incubation times (60–420 min). Then, the final concentration of MB was measured to evaluate the optimum time for removal of MB using MB-MIP membrane.

#### 3.3.5. Isotherm Study for Sorption of MB using MB-MIP Membranes

The sorption isotherm of MB-MIP membranes was studied at the optimum pH and time, where a piece of MIP membrane (0.012 g) was incubated in different concentrations of MB. Then, the filtrate was analyzed using the UV-visible spectrophotometer.

#### 3.3.6. Selectivity Study of MB-MIP Membranes

The binding of MB and some structurally related compounds such as methylene orange (MO) and fast green (FG) by MB-MIP membranes were studied. A piece of MIP membrane (0.012 g) was incubated in MB solution (20 mL, 10 ppm) and shaken with optimum pH, time and concentration. The filtrate was analyzed using a UV-visible spectrophotometer. Same procedure was adopted for MO and FG.

## 4. Conclusions

MB-MIP membranes were successfully prepared by hybridizing MB-MIP powders with CA and PSf polymer using an inversion phase process. The FTIR results confirmed the interaction between MB-MIP powders with both polymers. The SEM images of CA-MB-MIP and PSf-MB-MIP membrane proved that hybridization processes were successfully performed. The sorptions of MB on the MB-MIP membranes were optimized under alkaline conditions. MB sorption using the MB-MIP membranes can be described using pseudo second order and Freundlich isotherm models. Based on the results, the MB-MIP membranes are able to remove MB rapidly (within 60 min) with high binding capacity. The MB-MIP membranes were also proven selective towards MB when compared to MO and FG. 
